# Supplementary material for: Transurethral Resection of Non-Muscle Invasive Bladder Tumors Combined with Fluorescence Diagnosis and Photodynamic Therapy with Chlorin e6-Type Photosensitizers
Source: J Clin Med. 2021 Dec 31;11(1):233. doi: 10.3390/jcm11010233 (PMC8745686; doi:10.3390/jcm11010233)
Supplement: Supplementary file 1 [file jcm-11-00233-s001.zip › jcm-1454411-supplementary.pdf]

Supplementary Material to the manuscript by Andrey V. Kustov *et al*.

"Transurethral resection of non-muscle invasive bladder tumors combined with fluorescence diagnosis and photodynamic therapy with chlorin e6-type photosensitizers"

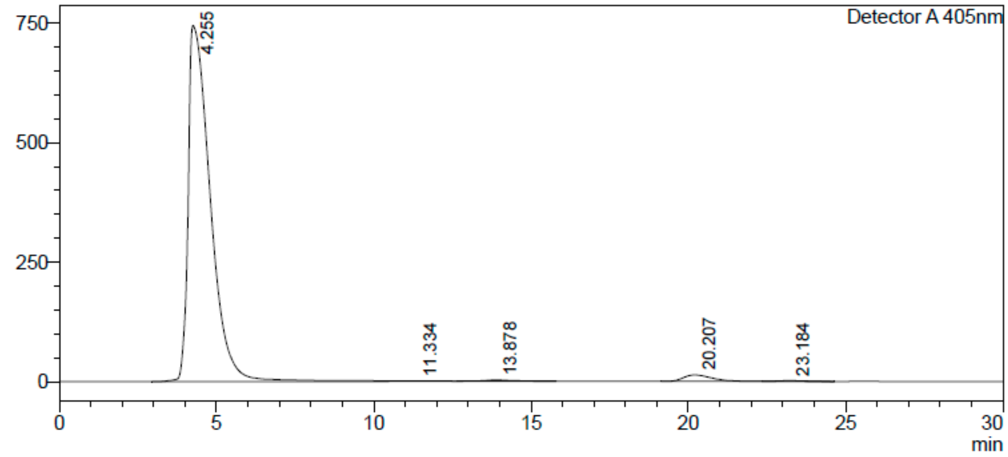

<Peak Table>

Detector A 405nm

Peak#	Ret. Time	Area	Height	Area%
1	4.255	36652676	745643	97.186
2	11.334	8953	172	0.024
3	13.878	150074	2505	0.398
4	20.207	830827	13336	2.203
5	23.184	71446	1219	0.189
Total		37713976	762874	100.000

(**a**)

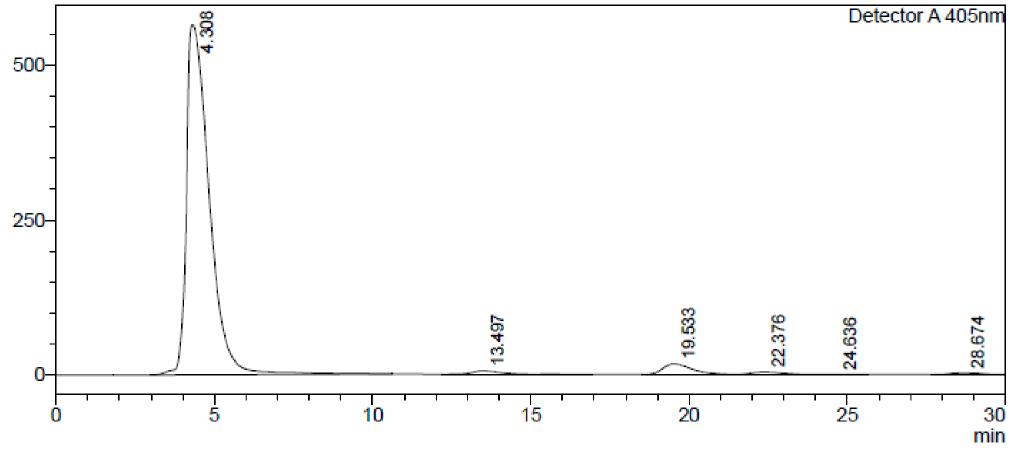

<Peak Table>

Detector A 405nm

Peak#	Ret. Time	Area	Height	Conc.	Unit	Mark	Name
1	4.308	28378165	565674	93.848			
2	13.497	346697	5416	1.147		S	
3	19.533	1077126	16984	3.562			
4	22.376	255833	4018	0.846		V	
5	24.636	29365	466	0.097		V	
6	28.674	151111	2448	0.500			
Total		30238298	595006				

(**b**)

Figure S1. The HQLC analysis of "Fotoran e6" (**a**) and "Fotoditazin" (**b**) purity with a PLC Shimadzu Prominence equipped with the SPD-20 A detector.

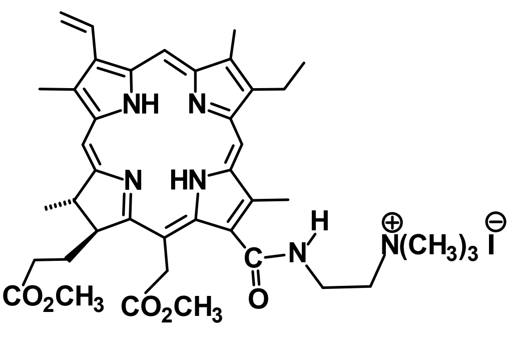

(**a**)

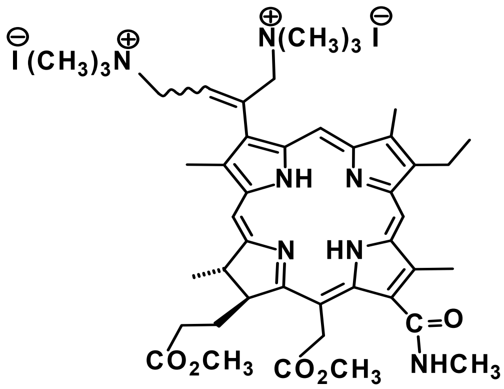

(**b**)

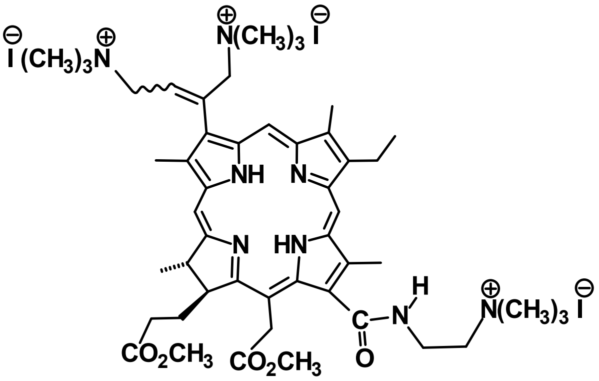

(**c**)

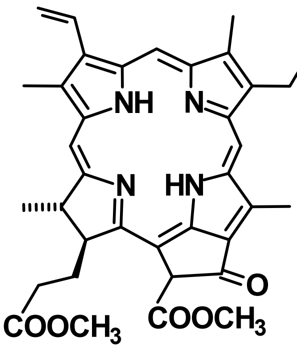

(**d**)

Figure S2. Molecular structures of PSs mentioned in Table 1 of the main text: (**a**) 13(1)-N-(2-N'N'N'-trimethyl-ammonioethyl iodide)amide chlorin e6 15(2),17(3)-dimethyl ester (Monocationic chlorin), (**b**) 3(1),3(2)-*bis*-(N,N,N-trimethylaminomethyl iodide) chlorin e6 13(1)-N-methylamide 15(2),17(3)-dimethyl ester (Dicationic chlorin); (**c**) 3(1),3(2)-*bis*-(N,N,N-trimethylaminomethyl iodide) chlorin e6 13(1)-N'-(2-N'',N'',N''-trimethylammonioethyl iodide)amide 15(2),17(3)-dimethyl ester (Tricationic chlorin); (**d**) pheophorbide *a* 17(3)-methyl ester (methylpheophorbide *a*).

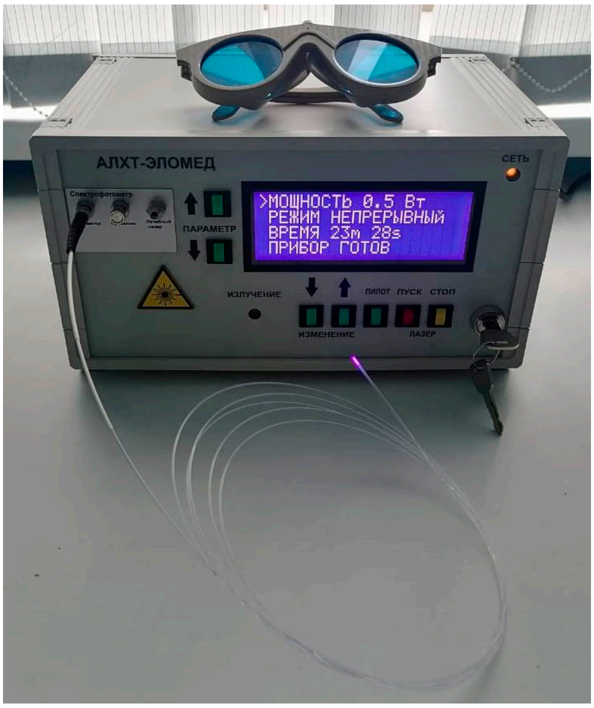

(**a**)

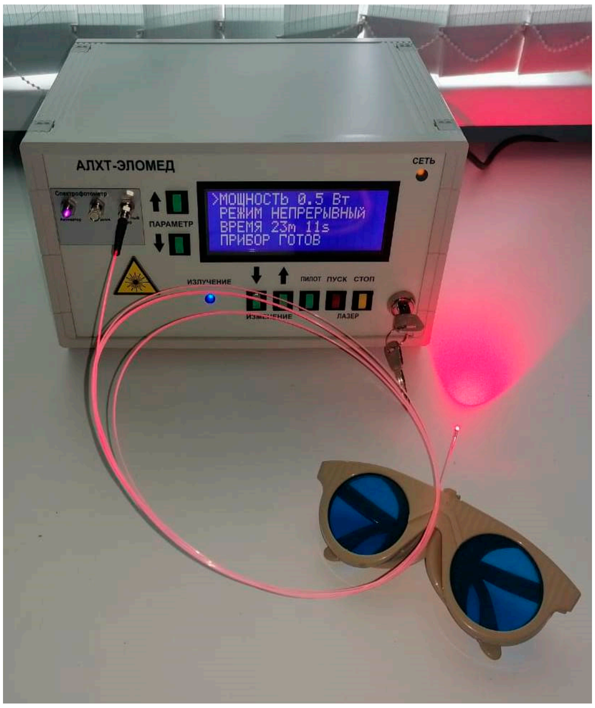

(**b**)

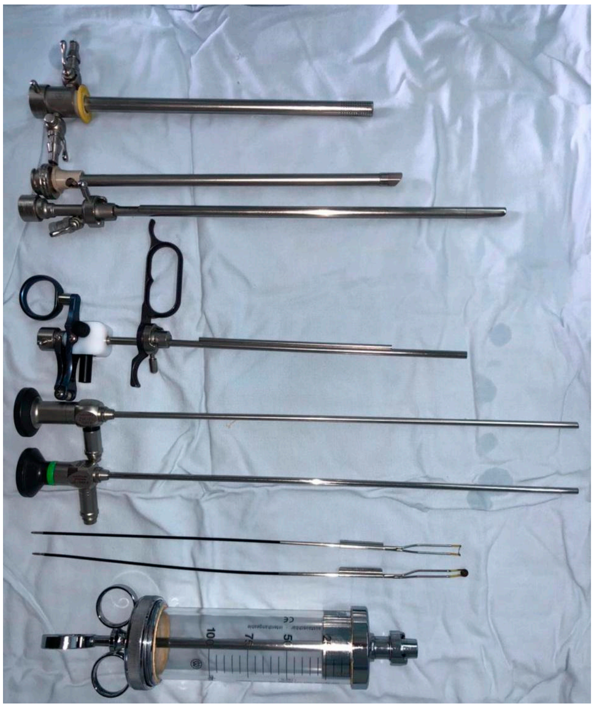

(**c**)

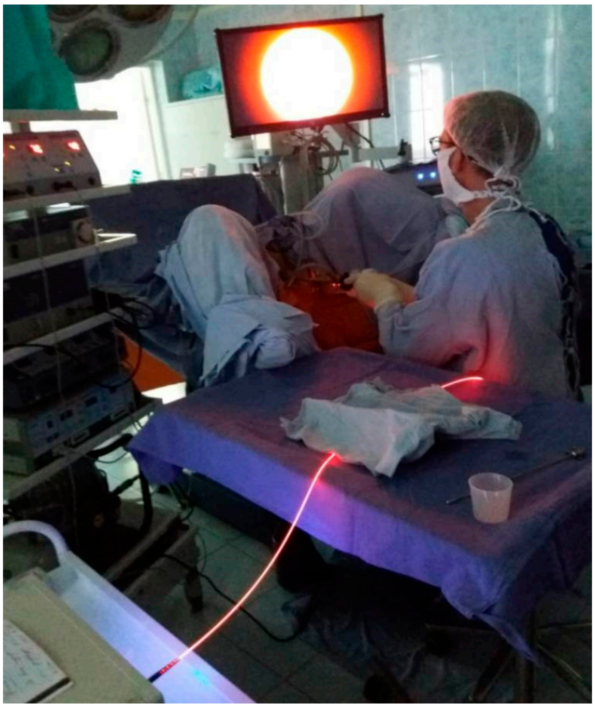

(**d**)

Figure S3. Activated "ALXT-Elomed" laser at 400 nm with a cylindrical diffuser for FD (**a**) and at 660 nm with a mictolens for PDT (**b**); a standard set ofinstruments for TURB (**c**) and PDT of the tumor bed performed by Oleg A. Privalov at IRCH (**d**).

Video S1 of TURB+FD+PDT is available on-line at https://www.youtube.com/watch?v=R73CVzE-owQ&feature=emb_imp_woyt or https://elomed.ru/lazer-dlya-urologii.html

Calculation of irradiation time for the PDT treatment

Irradiation time both of the tumor bed and the whole bladder was calculated in a similar way as described several times [8,24,28]. Briefly, after fulguration, the size of the tumor bed was estimated *via* a 5-Fr catheter with the length markings inserted into the central axis of the bladder. The radius of a light spot was chosen to be 0.4–0.5 cm larger than the radius of the tumor bed. The square of the light spot was calculated as follows

$$S = \pi \cdot r^2 = \pi \cdot (d/2)^2 \quad \text{(S1)}$$

The fluence rate (P_S, W/cm^2) was calculated by

$$P_S = P/S, \quad \text{(S2)}$$

and irradiation time (τ, sec) as

$$\tau = D/P_S, \quad \text{(S3)}$$

where P is the output beam power (W) and D is the necessary light dose (J/cm^2).

To compute time of diffuse irradiation of the whole bladder, the spherical bladder model was applied. The bladder radius was calculated from its volume as follows

$$r = \sqrt[3]{\frac{3}{4} V / \pi} \quad \text{(S4)}$$

The internal area of the bladder (S_{ia}, cm^2) and irradiation time were estimated by the following way

$$S_{ia} = 4 \cdot \pi \cdot r^2 \quad \text{(S5)}$$

$$\tau = D \cdot S_{ia}/P \quad \text{(S6)}$$

References

8. Lee, J.Y.; Diaz, R.R.; Cho, K.S.; Lim, M.S.; Chung, J.S.; Kim, W.T.; Ham, W.S.; Choi, Y.D. Efficacy and safety of photodynamic therapy for recurrent, high grade nonmuscle invasive bladder cancer refractory or intolerant to bacille Calmette-Guérin immunotherapy. *J. Urol.* **2013**, *190*, 1192–1199. https://doi.org/10.1016/j.juro.2013.04.077.

24. Kustov, A.V.; Berezin, D.B.; Strelnikov, A.I.; Lapochkina, N.P. *Antitumor and Antimicrobial Photodynamic Therapy: Mechanisms, Targets, Clinical Laboratory Research. A Practical Guide*; Gagua, A.K., Ed.; Largo: Moscow, Russia, 2020; pp. 1–108. (In Russian)

28. Chin, W. Photosensitizing Effects Chlorin e_6—Polyvinylpyrrolidone for Fluorescence Guided Photodynamic Therapy of Cancer. Ph.D. Thesis, Singapore National University, Singapore, 2009.
